# DAMPs and NETs in Sepsis

**DOI:** 10.3389/fimmu.2019.02536

**Published:** 2019-10-30

**Authors:** Naomi-Liza Denning, Monowar Aziz, Steven D. Gurien, Ping Wang

**Affiliations:** ^1^Center for Immunology and Inflammation, Feinstein Institutes for Medical Research, Manhasset, NY, United States; ^2^Elmezzi Graduate School of Molecular Medicine, Manhasset, NY, United States; ^3^Department of Surgery, Donald and Barbara Zucker School of Medicine at Hofstra/Northwell, Manhasset, NY, United States; ^4^Department of Molecular Medicine, Donald and Barbara Zucker School of Medicine at Hofstra/Northwell, Manhasset, NY, United States

**Keywords:** DAMPs (damage-associated molecular patterns), NETs (neutrophil extracellular traps), sepsis, HMGB1 (high-mobility group box 1), CIRP, cold-inducible RNA-binding protein, histone, neutrophils

## Abstract

Sepsis is a deadly inflammatory syndrome caused by an exaggerated immune response to infection. Much has been focused on host response to pathogens mediated through the interaction of pathogen-associated molecular patterns (PAMPs) and pattern recognition receptors (PRRs). PRRs are also activated by host nuclear, mitochondrial, and cytosolic proteins, known as damage-associated molecular patterns (DAMPs) that are released from cells during sepsis. Some well described members of the DAMP family are extracellular cold-inducible RNA-binding protein (eCIRP), high mobility group box 1 (HMGB1), histones, and adenosine triphosphate (ATP). DAMPs are released from the cell through inflammasome activation or passively following cell death. Similarly, neutrophil extracellular traps (NETs) are released from neutrophils during inflammation. NETs are webs of extracellular DNA decorated with histones, myeloperoxidase, and elastase. Although NETs contribute to pathogen clearance, excessive NET formation promotes inflammation and tissue damage in sepsis. Here, we review DAMPs and NETs and their crosstalk in sepsis with respect to their sources, activation, release, and function. A clear grasp of DAMPs, NETs and their interaction is crucial for the understanding of the pathophysiology of sepsis and for the development of novel sepsis therapeutics.

## Introduction

Sepsis is common and deadly; 30–50% of patients suffering an in-hospital mortality have sepsis. In the United States, sepsis affects 1.7 million adults annually resulting in more than 250,000 deaths (1, 2). It is estimated that, worldwide, sepsis impacts 30 million people per year and leads to 6 million deaths (3). Until recently, sepsis was defined as the systemic inflammatory response syndrome (SIRS)—hypo or hyperthermia (>38°C or <36°C), increased heart rate and respiratory rate and increased or decreased white blood cell count- in the presence of an infection. Sepsis with organ dysfunction was severe sepsis and fluid-refractory hypotension was septic shock (2). New guidelines, called Sepsis-3, established new definitions of sepsis, defining sepsis as “life threatening organ dysfunction caused by dysregulated host response to infection” (2). Organ dysfunction, as recommended by Sepsis-3, is defined clinically as changes of 2 points or more on the Sequential [Sepsis-related] Organ Failure Assessment (SOFA). The most severe subset of sepsis—septic shock- is defined as “sepsis in which underlying circulatory and cellular metabolism abnormalities are profound enough to substantially increase mortality” (2).

Sepsis arises from the body's exaggerated immune response to infection (4). Based on the “germ theory” of disease (5), it was initially thought that the inflammation, organ injury, and death that follows an infection were solely due to the body's response to microbial products, such as pathogen-associated molecular patterns (PAMPs) (6). PAMPs are recognized by pattern recognizing receptors (PRRs) expressed on immune-reactive cells (7). Numerous studies have been published to demonstrate the role of PAMPs and PRRs in activating the immune system in sepsis (4, 6). During the last several decades, subsequent studies have identified damage-associated molecular patterns (DAMPs). DAMPs are host nuclear or cytoplasmic non-microbial molecules which, when released from the cell following tissue injury, serve as potent activators of the immune system initiating and perpetuating a non-infectious inflammatory response to cause systemic inflammation, organ injury, and death (8–10). Like PAMPs, DAMPs are also recognized by PRRs and utilize the same signal transduction machinery to activate the immune system (6, 11). Clinically, sepsis severity has been shown to correlate with DAMPs; studies have shown that increased serum levels of DAMPs including high mobility group box 1 (HMGB1), extracellular cold-inducible RNA-binding protein (eCIRP), and H3 correspond with increased with disease severity (12–14). This review describes several well-known DAMPs, details the mechanisms of their release and actions, and describes therapeutic strategies that target DAMPs in sepsis.

Neutrophils are the most abundant leukocytes in the body and serve as the first line of defense against infection (15). The effector function of neutrophils is mediated through phagocytosis, reactive oxygen species (ROS), and protease dependent killing of ingested pathogens. In addition, activated neutrophils release neutrophil extracellular traps (NETs)—webs of DNA and anti-microbial proteins designed to kill pathogens (16, 17). The discovery of NETs provided new insights into neutrophil effector function. However, numerous studies have also revealed the detrimental role of NETs in sepsis (18). Homeostasis in regards to NETs requires the interplay between their beneficial bactericidal properties and the hyperstimulation of immune cells by the DNA and proteins contained within NETs that results in inflammation and tissue injury in sepsis.

**Figure 1:**
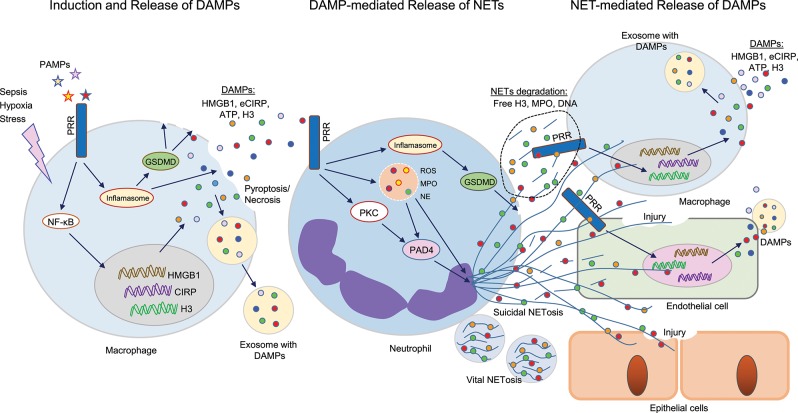
Cross talks between DAMPs and NETs in sepsis. Sepsis or hypoxia activates immune reactive cells, including macrophages, and neutrophils. In bacterial sepsis, PAMPs interact with PRR on macrophages to activate NF-κB, leading to increased expression of DAMPs (HMGB1, CIRP, H3) at transcriptional and translational levels. These intracellular DAMPs are then released extracellularly through different mechanisms, such as inflammasome-mediated GSDMD activation, which causes increased membrane pore formation to release intracellular DAMPs, or pyroptosis-, necroptosis-, or exosome-mediated pathways. These DAMPs can in turn recognize PRR on surrounding neutrophils and activate PAD4, GSDMD to promote NET formation. NETs components such as H3, MPO, or DNA can further activate immune cells and endothelial cells to release increased levels of DAMPs to augment the inflammatory cascade. In epithelial cells, extracellular histones derived from NETs promote cell/tissue injury, resulting in increased severity of ALI. DAMPs, damage-associated molecular patterns (DAMPs); NETs, neutrophil extracellular traps; PAMPs, pathogen-associated molecular patterns; PRR, pattern recognizing receptors; GSDMD, gasdermin D; HMGB1, high mobility group box 1; CIRP, cold-inducible RNA-binding protein; PAD4, peptidoglycan arginine deiminase 4; ALI, acute lung injury.

A number of review articles have been published demonstrating the individual role of DAMPs or NETs in sepsis (6, 19, 20). In sepsis, DAMP mediated signaling fuels pro-inflammatory cytokine and chemokine production by macrophages and other immune cells. This, in turn, leads to excessive neutrophil infiltration into the tissue. Activated neutrophils produce reactive oxygen species (ROS), inducible nitric oxide synthase (iNOS), and NETs which contain noxious molecules, leading to tissue inflammation and injury in sepsis. In this review, we focus on DAMPs, NETs, and explore their interplay during sepsis (Figure 1). We also discuss some of the therapeutic interventions targeting both DAMPs and NETs in experimental sepsis (Table 1).

**Table 1:** Therapeutic outcomes by targeting DAMPs and NETs in sepsis.

**DAMPs/NETs**	**Strategies**	**Outcomes**	**References**
eCIRP	CIRP^−/−^ mice; Anti-CIRP Ab; C23	Decreased organ injury markers (AST, ALT, LDH), decreased cytokines and chemokines, protected from lung injury including decreased MPO levels, neutrophil infiltration, and cellular apoptosis in lungs.	(4)^a^^b^, (43)^a^^,^^b^, (68)^a^, (69)^a^
HMGB1	Anti-HMGB1 Ab; Zingerone; HMGB1-antagonsits interacting with RAGE; small molecule inhibitors of HMGB1; sodium sulfonate derivative of tanshinone IIA (TSNIIA-SS); synthetic molecules including nafamostat mesylate and gabexate mesylate; peptide inhibitors including vasoactive intestinal peptide, pituitary adenylate cyclase-activating polypeptide (PACAP), and urocortin	Increased survival after endotoxemia and CLP, improved cytokine profile after CLP sepsis, inhibited LPS-induced HMGB1 secretion, reduced vascular permeability, reduced expression of cellular adhesion molecules, reduced sepsis-mediated liver injury, reduced LPS-mediated cytokine release and lung injury.	(56)^a^^,^^b^, (57)^a^, (25)^a^^b^
Histone	Anti-histone ab; Activated Protein C	Increased survival in LPS, TNF-α, and CLP sepsis, rescued from lethality in E. coli infusion, attenuated cardiac injury and dysfunction in sepsis.	(71)^c^, (73)^b^
ATP	P2X7 receptor blockade ^+/−^ adenosine A_2A_ receptor stimulation; ATP hydrolase (apyrase)	Prevented tissue damage, apoptosis, and cytokine production in the liver of mice after CLP, reduced cytokines, prevented mitochondrial damage, reduced apoptosis, reduced intestinal barrier disruption, increased survival.	(89)^a^, (90)^a^
NETs	DNAse I; PAD4^+/−^ mice; CL-Amidine; Anti-citrullinated histone 3 Ab	Reduced lung injury and increased survival in a pneumonia model, reduced NETs and improved survival in CLP sepsis.	(135)^a^^b^, (180)^a^, (185)^a^

a *Rodent*,

b *Human*,

c *Non-human primates*.

## DAMPs

DAMPs were first proposed as part of the “Danger Theory” by Polly Matzinger in the mid 1990's as an initial explanation for the robust inflammatory response elicited in response to sterile inflammation, which could not be explained solely by the self vs. non-self-hypothesis of the time (8). Intracellularly, DAMPs are hidden from view of the innate immune system. After tissue injury, caused by either sterile or infectious insults, they are released extracellularly to activate the immune system and resultant pro-inflammatory cascades (34). As discussed above, DAMPs are thus defined as endogenous molecules that can initiate and potentiate a non-infectious inflammatory response (8). In addition to their role in sepsis, as is discussed in the rest of this article, the release of DAMPs is critical to the development of sterile inflammation including inflammation that occurs secondary to organ ischemia and reperfusion injuries (35–37), non-infectious inflammatory liver diseases such as non-alcoholic fatty liver disease (38), or the sterile inflammation associated with aging (39).

Allowing the evolution of the Danger Theory from an abstract concept to a concrete entity, probably the first DAMP identified was HMGB1 (40, 41). Other DAMPs include histones, ATP, uric acid, DNA, mitochondrial DNA, and IL-33 (42). Recently, eCIRP has been identified as a newly discovered DAMP (43, 44). Although numerous endogenous molecules have been identified as inflammation-causing DAMPs, here we briefly review a selective group of DAMPs which have been strongly implicated in sepsis.

## HMGB1

HMGB1 is a highly conserved protein expressed in all mammalian cells (21). HMGB1 as a DAMP causing sterile inflammation was discovered in 1999 (41). HMGB1 can be released actively via cytoplasmic vesicles or passively from necrotic cells. Active release is mediated by several pathways; JAK/STAT-1 mediated acetylation is responsible for the initial HMGB1 translocation from the nucleus to the cytoplasm, while extracellular release is partially mediated by double-stranded RNA-activated protein kinase R (PKR)/inflammasome-mediated pyroptosis (45). While passive release after necrotic cell death is rapid, active HMGB1 release is much slower. HMGB1 levels reach a plateau approximately 16–32 h after the onset of endotoxemia (46). HMGB1 related signaling is modulated by the redox state of its three cysteines (numbers 23, 45, and 106) (47, 48). Once released into the extracellular space, HMGB1 activates innate immune cells to propagate pro-inflammatory signaling cascades (49). HMGB1 induces recruitment of neutrophils to the site of tissue injury (50). HMGB1 binds to other PAMPs, including DNA (51), LPS (52), and lipoteichoic acid (53), potentiating their inflammatory responses. HMGB1 has been shown to bind to numerous cell surface receptors, including but not limited to receptor for advanced glycation end products (RAGE), TLR2, TLR4, TLR9, and triggering receptor expressed in myeloid cells 1 (TREM-1) (49, 54). After binding to these receptors, it activates macrophages and endothelial cells, stimulating the production of proinflammatory chemokines, cytokines, and endothelial adhesion molecules (49). HMGB1 is elevated in patients with sepsis (12, 55), and dozens of studies have demonstrated that targeting HMBG1 improves outcomes in sepsis (24, 25, 56, 57).

## eCIRP

Extracellular CIRP is an 172-amino acid RNA chaperone protein (26, 58–60) that was previously identified as a DAMP in 2013 (43). It is a cold shock protein, originally recognized as a protein that suppresses mitosis and promotes cell differentiation in the setting of hypothermia (61). It is upregulated by hypothermia, hypoxia, and oxidative stress, such as UV irradiation. In addition to passive release during necrotic cell death, in times of cellular stress (like the aforementioned hypothermia, hypoxia, or oxidative stress), CIRP can translocate from the nucleus to cytoplasmic stress granules; from these, it is released to the extracellular space (62). After eCIRP binding to its receptor, the TLR4-myeloid differentiation factor 2 (MD2) receptor complex (43), activation proceeds through the TLR4/MyD88/NF-κB pathway (63) to stimulate the release of pro-inflammatory cytokines TNF-α and HMGB1 from macrophages (43). Furthermore, during sepsis, hemorrhage or ischemia-reperfusion (I/R) injury, CIRP is released extracellularly and leads to organ injury (36, 43). Elevated plasma levels of eCIRP have been independently correlated with a poor prognosis in patients with sepsis (13).

eCIRP as a DAMP has been demonstrated in several cell types including macrophages, lymphocytes, and neutrophils in the context of cellular activation, cytokine and chemokine production and neutrophil extracellular trap (NET) formation (44). eCIRP has also been shown to stimulate the Nlrp3 inflammasome, cause endoplasmic reticulum (ER) stress, and induce pyroptosis in lung endothelial cells (EC) (64, 65). eCIRP is associated with acute lung injury (ALI). Healthy mice injected with recombinant murine (rm) CIRP develop ALI via macrophage, neutrophil, and EC activation, and cytokine production in the lungs (65). Beneficial outcomes have been seen in CIRP^−/−^ mice or CIRP inhibition in murine models of renal, intestinal, and hepatic I/R injury (36, 66, 67). CIRP^−/−^ mice are protected from sepsis and ALI (64, 65). In an animal models of adult or neonatal sepsis, treatment with a polyclonal anti-CIRP antibody or a CIRP-derived inhibitory peptide prolonged survival and attenuated organ injury (43, 68, 69).

## Histones

Histones are highly basic proteins that are located mainly in the nucleus. In humans, histone H2A, H2B, H3, and H4 form a complex with DNA, called a nucleosome. The nucleosome regulates gene transcription and facilitates efficient higher-order chromatin compaction (22). However, histones play proinflammatory functions upon their release from the nucleus into the extracellular environment (23). Histone release from cells can occur passively after cellular necrosis or as part of an active process such via NETosis (70). In 2009, Xu et al. demonstrated that histones were cytotoxic when added to cultured endothelial cells (71). *In vivo*, intravenous injection of histones in mice was lethal, whilst anti-histone antibodies were found to reduce mortality in murine models of LPS endotoxemia, TNF-α, or cecal ligation, and puncture experimental models of murine sepsis (71). Xu subsequently demonstrated that the injection of sublethal doses of histones resulted in high levels of the cytokines TNF-α, IL-6, and IL-10, a phenomenon which did not occur when TLR4^−/−^ mice were used. Conversely, TLR2^−/−^ mice maintained their hyperinflammatory profiles after histone injection (72). However, using specific TLR-transfected HEK cells, histones signaling was transduced via both TLR4 and TLR2 (72). Histones have also been shown to bind to TLRs in cardiomyocytes where they alter levels of regulatory proteins and potentiate sepsis-induced cardiomyopathy (27). The impact of histones has also been investigated in human sepsis. *Ex-vivo* administration of serum from septic patients directly induced cardiomyocyte death; this effect was abolished by anti-histone antibody (73). Histone levels in septic patients are significantly increased and, like in murine models, appear to cause cellular injury in a TLR4 dependent method (14).

## Cell Free DNA

In the extracellular space, deoxyribonucleic acid (DNA) can serve as a DAMP. Apoptosis, necroptosis, NETosis, and pyroptosis can all contribute to the release of nuclear contents into the extracellular space (74). Cell free DNA in plasma is elevated in patients with severe sepsis or septic shock when compared to patients without these diagnoses (28), and increased levels of cell free DNA in the plasma of septic patients has been linked to increased mortality during sepsis (75).

Viral, bacterial, and even host cell free DNA can all function as a DAMP and initiate pro-inflammatory cascades (74, 76). Additionally, mitochondrial DNA (mtDNA) has been proven to be a DAMP; it is released into the circulation during trauma or sepsis (77, 78). mtDNA has been shown to cause TNF-α secretion by mouse splenocytes and IL-1β release from bone marrow-derived macrophages (79). In addition to promoting the release of proinflammatory cytokines, DNA has been shown to prolong the lifespan of neutrophils. Neutrophils stimulated with either purified bacterial or mitochondrial DNA demonstrated increased viability compared to controls (78). Excessive neutrophil accumulation in tissues has been associated with poor outcomes in sepsis (80).

Viral, bacteria, host cell free DNA, and mtDNA can all act via the TLR9 receptor (74), which is located intracellularly in endosomes (81). It is important to recognize the spatial relationship of DNA that acts as an immunomodulatory molecule and the TLR9 receptor. TLR9's intracellular location requires that nuclear DNA molecules that are released into the extracellular space by NETosis, apoptosis and other forms of cell death need to be translocated intracellularly in recipient cells in order to activate the TLR9 receptor (74). Besides TLR9, intracellular DNA can trigger other alarmin sensors such as cyclic guanosine monophosphate-adenosine monophosphate synthase (cGAS), absent in melanoma 2 (AIM2), interferon-inducible protein 16 (IFI16), and stimulator of interferon genes (STING), all of which lead to the initiation of immune responses (74).

## ATP

ATP is a nucleotide that, in times of homeostasis, is generated mainly within mitochondria during the tricarboxylic acid cycle and from the respiratory chain. ATP is also produced in the cytoplasm during glycolysis (82). ATP is released actively from dying cells during apoptosis, and passively during necroptosis and cellular necrosis (38, 83). Although some extracellular ATP is beneficial, as it functions as a chemoattractant recruiting phagocytic cells to the site of tissue damage, extracellular ATP is also detrimental, binding to ionotropic P2X receptors (P2XR) (84). P2XR channel opening results in increases in intracellular calcium, which activates the p38 MAPK pathway, activating the inflammasome with the associated caspase-1 activation and release of pro-inflammatory cytokines IL-1β and IL-18 (84–86). Elevated ATP levels in the plasma of septic patients interfere with neutrophil function and signaling, resulting in an excessive and uncoordinated neutrophil activation (87). Excessive extracellular ATP has also been associated with T cell suppression in sepsis (88). Reduction in the extracellular levels of ATP has proven to be an effective method of attenuating sepsis severity in some murine models of sepsis. Removal of extracellular ATP to decrease activation of the P2X7 receptor by CD39 has been shown to attenuate sepsis-induced liver injury (89). Treatment with apyrase, an ATP hydrolase that removed extracellular ATP, protected mice against a lethal LPS challenge and resulted in a reduction of serum cytokines (90).

## Molecules That May or May Not be DAMPs

**Figure 2:**
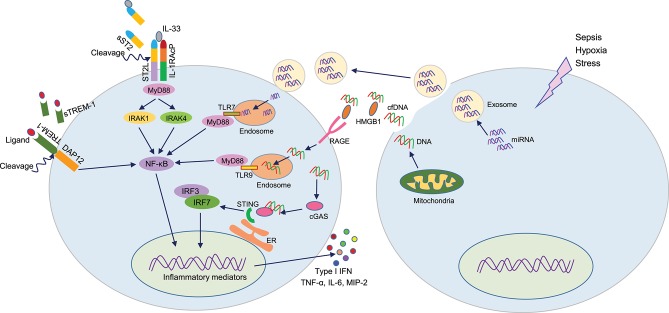
DAMPs or not DAMPs? In sepsis, extracellular motifs of several receptors like TREM-1, ST2 are cleaved by matrix metalloproteinases, leading to increased accumulation of truncated receptors in the blood. These soluble receptors serve as decoy molecules to recognize their ligands, thereby modulating respective intracellular signal transduction. During sepsis, cells release miRNA or cfDNA through exosomes or passively. Extracellular miRNAs can enter into adjacent cells and recognize endosomal TLR7 to induce inflammation. cfDNA can recognize HMGB1, and this protein-DNA complex is then recognized by the RAGE receptor and become internalized. Intracellular cfDNA then can activates endosomal TLR9 or STING to activate the production of pro-inflammatory mediators. DAMPs, damage-associated molecular patterns (DAMPs); TREM-1, triggering receptor expressed on myeloid cells-1; HMGB1, high mobility group box 1; cfDNA, cell-free DNA; STING, stimulator of interferon genes.

Several endogenous molecules located intracellularly or on the cell surface are released into the circulation and serve as diagnostic and prognostic markers in various inflammatory diseases (4, 29). These molecules include components of the extracellular matrix (ECM) like collagen, fibrinogen, and laminin and shredded cell surface receptors, such as soluble ST-2(30), a member of the interleukin 1 receptor family, sMD2(91), sTREM-1(92), microRNAs (93), exosomes (94), and vesicles (95). However, it is not clear whether these and similar molecules should be classified as DAMPs (Figure 2). DAMPs are frequently released from cells following necrosis, pyroptosis or apoptosis, however the ECM, shredded receptors, exosomes, micro-vesicles are released into the extracellular environment without cell lysis. Conversely, mtDNA and cell-free DNA are classified as DAMPs and are released in both suicidal and vital NETosis, meaning a molecule can be classified as a DAMP without cell lysis first occurring. Many DAMPs undergo structural modification (96, 97) e.g., oxidation, reduction, acetylation, phosphorylation, or cleavage after release into the circulation. Conversely, it is not known whether the shredded receptors or exosomal molecules undergo post release modification in the extracellular milieu. Extracellularly, DAMPs play largely pro-inflammatory roles, while the secreted proteins, cleaved receptors, exosomes and vesicles are not always pro-inflammatory and are not necessarily responsible for excessive inflammation (98). Cell surface proteins that are shed have diverse functions and include chemokines, cytokines, adhesion molecules, growth factors, and their receptors (99).

The shedding process of these proteins regulates the density of cell surface receptors, the release of factors that serve as agonists, and the release of soluble receptors that can function as antagonists (100). Cleaved receptors such as sTREM-1 acts as a decoy receptor, sequestering TREM-1-ligands and dampening TREM-1 activation (101, 102). Soluble ST-2 serves as an antagonist for IL-33 to control excessive innate immune response (103). Exosomes, macrovesicles, and microparticles are enriched in pro- and anti-inflammatory molecules, therefore they may play dual roles in sepsis. LPS-challenged macrophages have been shown to release histone-coated microvesicles to cause inflammation (104). Exosomes released from alveolar macrophages during hemorrhagic shock have been shown to promote necroptosis (105). By contrast, exosomes filled with anti-inflammatory molecule milk fat globule-EGF-factor-8 (MFG-E8) were shown to be beneficial in reducing markers of inflammation in sepsis and improving survival (106). Cleaved receptors or exosomes often directly serve as chemoattractants (107), but the ability of DAMPs to directly serve as a chemokine are not as well studied.

Excess production and release of ECMs may cause tissue fibrosis, abnormal cell proliferation, migration and inflammation (108). Receptor protein cleavage occurs due to the actions of matrix metalloproteinases (MMPs), disintegrins, and metalloproteinases (ADAMs) which are upregulated during inflammation (109). The exosomes and microvesicles are released from the cells through pore formation in the plasma membrane by caspase-mediated GSDMD or by a budding out process (110). The release of excess amount of exosomes and microvesicles are correlated with an increased release of DAMPs, allowing the possibility that exosomes and microvesicles may be a mechanism of DAMP release in sepsis (111). Exosomes and microvesicles may also serve as a means to maintain cell to cell communication; they have the ability to enter into adjacent cells and modulate function. Extracellular microRNAs levels are increased in various inflammatory conditions and may serve as diagnostic markers (112). Studies have shown that extracellular microRNA plays a pro-inflammatory role following its re-entry into macrophages and activation of the endosomal TLR7 receptor to produce TNF-α and IL-6 (113).

More studies on these molecules will help elucidate their pathophysiological role in sepsis and other inflammatory conditions. This information will aid in clarification of these molecules as DAMPs or non-DAMPs.

## NETs

Neutrophils are phagocytic cells; they predominantly defend against pathogens either by engulfing the offending cell and destroying it via oxidant- or protease-dependent mechanisms or by the secretion of anti-microbial peptides (114). This classical understanding of neutrophil function was found to be incomplete after the discovery of a third effector function of neutrophils in 2004, the release of NETs (17). NETs are web-like chromatin based structures that are released into the extracellular environment to aid in pathogen clearance, but they have also been implicated in excessive inflammation with resultant tissue damage, potentiation of autoimmunity, and promotion of vascular thrombosis (16). NETosis is a form of cellular death in which neutrophils decondense their nuclear chromatin and DNA into the cytoplasm. Chromatin and DNA mix with granule-derived antimicrobial peptides and are extruded into the extracellular space (115). NETosis can be induced in many ways; one of the most well-described is phorbol myristate acetate (PMA), a protein kinase C (PKC) activator (116).

NETs contain proteins from azurophilic granules e.g., neutrophil elastase (NE), myeloperoxidase (MPO) and cathepsin G; proteins from secondary and tertiary granules e.g., lactoferrin, and gelatinase; and nuclear proteins e.g., histones H1, H2A, H2B, H3, and H4 (117). Detection of NETs has proved challenging due their fragile structure, timing of NET formation and turnover, and ubiquitous presence of DNase I. Several tools to assay NETosis have been reported: these include microscopy (118), flow cytometry (119, 120), ImageStream^®^ (121), and ELISA (122). The ability to detect NETs precisely is paramount to studying the disease pathophysiology associated with NETosis.

## Mechanism of NET Formation

The first reported descriptions of NETs demonstrated that neutrophils stimulated with PMA, IL-8 or LPS released NETs (17). Subsequent studies have revealed a wide range of stimuli including bacteria, virus, fungi, yeast, parasites, and concanavalin A are capable of inducing NET formation (20). In addition, NETs are upregulated in various cancers, including pancreatic cancer, through receptor for advanced glycation end products (RAGE)-dependent and neutrophil autophagy mediated pathways (123). The induction of NETosis by various DAMPs will be discussed in the later part of this article.

Two forms of NETosis have been described: suicidal NETosis, in which NET formation only occurs via neutrophil cell death and was described above, and vital NETosis where NETs are released without cell death (124). In suicidal NETosis, NADPH-dependent ROS production is a prerequisite. This leads to increased calcium influx and peptidyl arginine deaminase 4 (PAD4) activation, leading to chromatin decondensation. Elastase and MPO are also transported from the granules to the nucleus to cleave linker histone H1 and modify the core histones. MPO also intensifies chromatin decondensation, through the synthesis of hypochlorous acid. Finally, chromatin is released outside the cell through membrane pores and cellular lysis through the activation of a pore forming protein GSDMD (125, 126).

First described in 2012, vital NETosis results in the release of NETs without a loss in the integrity of the nuclear or plasma membrane (127). As such, neutrophils are able to survive the process and are still capable of normal neutrophil functions including phagocytosis. Unlike suicidal NETosis, vital NETosis does not require generation of ROS or activation of the Raf/MERK/ERK pathway (126). In contrast to the several hour time frame required for stimulation of suicidal NETosis, vital NETosis occurs quickly, usually within 5 to 60 min after neutrophil are stimulated (128). In vital NETosis, after neutrophil stimulation, typically via TLR or complement receptor for C3 protein ligand binding, the nuclear membrane morphology changes to allow vesicle budding. These vesicles, containing nuclear DNA, move through the cytoplasm to coalesce with the plasma membrane and are released extracellularly (118, 124, 126).

Besides the aforementioned types of NETosis, in 2009 it was reported that neutrophils are able to undergo vital NETosis using mitochondrial DNA (129). GM-CSF primed neutrophils, when activated via TLR-4 or complement factor 5a receptor stimulation, generated NETs containing solely mitochondrial DNA. NETosis facilitated release of mitochondrial DNA seems to be ROS-mediated (129). *In vivo*, NETs containing mitochondrial DNA have been found in the serum of individuals after trauma (130) and associated orthopedic surgery (131).

Several other mechanisms of NET formation have been reported. Carestia et al. demonstrated that activated platelets are able to amplify the amount of NETs released from neutrophils (132). This process seemed to depend on interaction between glycoprotein Ib (CD42) in platelets with β2 integrin (CD18) in neutrophils, as well as the release of von Willebrand Factor. Platelet triggered NETosis did not rely on NADPH oxidase or ROS generation, but was reduced when inhibitors of ERK, PI3K, or Src kinases were used (132). NET formation has been shown to depend on the activation of cell-cycle proteins CDK4/6; Cdk6^−/−^ neutrophils and mice showed impaired NET formation to several stimuli including PMA and C. albicans (133). The lipoxin pathway has been shown to reduce lung inflammation and acute lung injury after both infectious and sterile inflammation (134). Lefrancais et al. demonstrated that this pathway, through Fpr2 receptor signaling, is a potent modulator of NET formation. After intratracheal injection of methicillin-resistant Staphylococcus aureus (MRSA), Fpr2^−/−^ mice produced excessive NETs compared to wild type mice (135). Additional studies are needed focusing on the pathways behind these types of NET formation to determine the type of NETosis- suicidal or vital.

## Phenotypic and Function Diversities of Neutrophils and NET Formation

Neutrophils exhibit phenotypic and functional heterogeneity (136). Neutrophil heterogeneity has tremendous impact on NET formation. Neutrophils from diabetic patients are more likely to undergo NETosis than neutrophils from euglycemic patients (31). Neutrophils from pediatric patients with systemic lupus erythematosus also undergo increased NETosis as compared to their healthy counterparts (137). ICAM-1 (CD54) is mainly expressed on the endothelial cell surface (138). Following simulation of neutrophils with PAMPs or DAMPs, ICAM-1 expression in the neutrophils is dramatically increased (139–141). The ICAM-1^+^ neutrophils produce higher levels of NETs, probably because of increased ROS (140). However, the involvement of ICAM-1 or its ligand Mac-1 in the increased levels of NETs in these cells has not been elucidated. The relationship seems to be circular, with NETs inducing ICAM-1 in neutrophils and ICAM-1^+^ neutrophils producing increased quantities of NETs (142). ICAM-1^+^ neutrophils are found in increased concentrations in blood and lungs of humans and mice under inflammatory conditions (143–146).

It is still not clearly known which type of neutrophils- circulating or tissue resident-produce increased levels of NETs. Using density gradient centrifugation, circulating neutrophils can be separated into two layers- high density neutrophils (HDN) and low-density neutrophils (LDN) which co-localize with peripheral blood mononuclear cells (147). LDN are a heterogeneous population containing both immature and mature neutrophils and their functions differ depending on the inflammatory stimulus (148, 149). Interestingly, it has been demonstrated that LDNs have an increased proinflammatory profile as compared to other neutrophils with increased secretion of proinflammatory cytokines (150, 151) and an increased capacity to generate NETs (149, 152, 153).

Since the ROS pathway is essential for suicidal NETosis (125), it is logical that neutrophils that produce increased levels of ROS may produce excessive NETs. Although evidence is conflicting (154), Zhang et al. found that aged neutrophils (CXCR4^+^) produced both increased levels of ROS and increased amounts of NETs (155). It is also evident that human neutrophils are more prone to produce NETs compared to murine neutrophils (156, 157), indicating the role of specific surface markers in NETs production between these species. Overall, neutrophil heterogeneity may play a pivotal role in NET formation.

## Increased NET Formation in Sepsis

NETs are vital to pathogen clearance, but simultaneously NETs induce collateral damage to host tissues in sepsis (16). In 2007, Clark et al. described an interaction between platelets and neutrophils in sepsis, resulting in NET formation and enhanced bacterial trapping in blood vessels (158). Activation of TLR4 receptors on platelets lead to the binding of the platelets to neutrophils in the blood. These neutrophils were then activated and produced NETs. These results were recapitulated using the plasma from severely septic patients (158).

Sepsis often results in acute lung injury (ALI) (159). Lefrancais et al. demonstrated abundant NET formation in both murine models of severe bacterial pneumonia and ALI (135). Furthermore, when comparing NET levels in samples from critically ill human subjects they found higher levels of NETs in subjects with infectious etiology of acute respiratory distress syndrome (ARDS) as opposed to patients with cardiac-induced respiratory dysfunction. In addition, among patients with microbiologically confirmed pneumonia, plasma NET levels were higher in patients with ARDS than in patients without. Finally, there was a correlation between the severity of ARDS, mortality, and the serum level of NETs (135).

In a clinical study, the levels of neutrophil-derived circulating free DNA (cf-DNA/NETs) have been shown to directly correlate with multiple organ dysfunction score, sepsis-related organ failure assessment, leukocyte counts, and MPO levels (160). A 2018 study of 55 critically ill patients demonstrated rapid and sustained increases in the circulating levels of MPO-DNA complex in the serum, indicating NET formation in the early stages of sepsis. In this study, MPO-DNA complex levels were also correlated with the severity of organ dysfunction and 28-day mortality rates (161).

In opposition to these findings, impaired NET formation in neonates has been associated with relative immunodeficiency of human newborns (162). Czaikoski et al. found increased bacterial burden in the blood and decreased survival in a murine model of CLP in mice treated with DNase to prevent NET formation, however these effects were ameliorated by treatment with DNase plus antibiotics (163). Given that there are both hyper and hypodynamic phases of sepsis, the levels of NETosis at various stages in sepsis may impact the outcomes. This idea is supported by work done by Mai et al. (164). They found that when given early after induction of sepsis by CLP, DNase increased pro-inflammatory cytokines and worsened renal and pulmonary damage. However, when given at a later timepoint after CLP, DNase administration reduced IL-6 levels, increased levels of anti-inflammatory IL-10, and reduced organ damage and bacterial dissemination. It also increased survival after CLP (164).

Several studies have demonstrated that severe sepsis alters the neutrophil phenotype and hinders NETosis *ex vivo* (165, 166). However, it is not clear from these studies whether *in vivo* NET formation is impaired during sepsis. Further investigation will need to be done in this area.

## Detrimental Effects of NETs in Sepsis

During sepsis, neutrophil-endothelial interaction is increased to promote neutrophil infiltration into tissues (167). Neutrophil-endothelial cell (EC) interaction leads to increased NET formation; this increased NET formation is partially dependent on IL-8 released from activated EC (168). Prolonged co-culture of neutrophils with EC resulted in EC damage; this damage is attributed to NETs as co-incubation with either NAPDH oxidase inhibitors or DNase ameliorated this damage (168).

Recent studies demonstrated the crucial role of NETs in the pathogenesis of disseminated intravascular coagulation and intravascular thrombosis, both of which increase morbidity and mortality in sepsis (169–173). McDonald et al. found profound platelet aggregation, thrombin activation, and fibrin clot formation within NETs, implicating the NET–platelet–thrombin axis in the promotion of intravascular coagulation in sepsis. Inhibition of NETs during sepsis by DNase infusion reduced intravascular coagulation, improved microvascular perfusion, and reduced organ damage (172).

NETs have been detected in bronchoalveolar lavage samples from septic humans or canines with ARDS, indicating that, even after transmigration, neutrophils are capable of undergoing NETosis (174, 175). A recent study utilizing samples from different models of ALI in mice and from patients with ALI revealed increased levels of NETs and histones H3 and H4 in the bronchoalveolar lavage fluids (BALF) (176). Administration of the extracellular histones contained in NETs resulted in damage to alveolar epithelial cells and increased severity of ALI (176).

In addition to the damage inflicted by the DNA released during NETosis, enzymes released during NETosis also have a detrimental effect on the surrounding tissues. Neutrophil elastase, a key component of chromatin degranulation, has been show to increase permeability of alveolar epithelial cells by altering the actin cytoskeleton (177) and its inhibition has been demonstrated to be beneficial in animal models of inflammation and associated ALI (178, 179). Serine proteases released during NETosis have been shown to degrade surfactants which are vital in the clearance of inflammatory cells and residual inflammation after ALI (18). These findings clearly demonstrate that excessive NETs play detrimental role in sepsis.

## Therapeutic Strategies Targeting NETs in Sepsis

Therapeutic strategies aimed at NETs primarily target the DNA component- DNase is the most frequent treatment modality. DNase treatment reduced NETs, improving lung injury and survival in a murine model of pneumonia (135). Cl-Amidine, a PAD4 inhibitor, had no effect on the level of neutrophil-DNA complexes or the degree of lung inflammation in a murine pneumonia model (135) but Biron et al. found that Cl-Amidine prevented H3 citrullination, NET formation, and improved survival in a murine model of CLP-induced polymicrobial sepsis (180). Similarly, PAD4^−/−^ mice demonstrated decreased NETs and lung injury in the pneumonia model (135). However, these benefits were offset by an increased bacterial load and increased systemic inflammation. Therefore, Lefrancais et al. developed a mouse with a partial PAD4 deficiency (PAD4^+/−^) which demonstrated an improved survival curve (135). These findings support the notion that a there is a thin line for the amount of NETosis required to both prevent lung injury and maintain microbial control.

Chloroquine has also been effective as an early upstream inhibitor of NETs, decreasing NETosis and the associated hypercoagubility and improving survival in murine models of pancreatic adenocarcinoma (181) and acute pancreatitis (32). Activated protein C (APC) is a multifunctional protease with anti-inflammatory, anticoagulant, and cytoprotective properties (182). A recent study demonstrated that APC binds human leukocytes and prevents activated platelet supernatant or PMA from inducing NETosis. Additionally, they found that pretreatment of neutrophils with APC prior to induction of NETosis inhibited platelet adhesion to NETs (182). It should be noted however, that activated protein C has failed to have any impact on survival in large scale human clinical trials of patients with severe sepsis (183, 184). Li et al. demonstrated that antibodies neutralizing serum citrullinated Histone 3 could improve survival after a murine CLP model (185). These studies demonstrate that abrogating excessive NET formation can lead to beneficial outcomes in sepsis.

The early inhibitors of NETs such as chloroquine, PAD4 inhibitors, and APC are specifically targeted for controlling NET formation. By contrast, late inhibitors of NETs, such as DNase and anti-histone antibodies, can target extracellular DNA or histones regardless of their source. These molecules are also considered as DAMPs and can be released by a number of immune cells, in addition to their release from neutrophils. Therefore, the molecules/drugs that specifically control intracellular NET formation could be used as a more specific therapeutic regimen against NETs.

## Crosstalk Between DAMPs and NETs in Sepsis and Inflammation

Although the extracellular release of DAMPs and NET formation are both a byproduct of sepsis, there is increasing evidence of linkage between the two. The major components of NETs, i.e., DNA, histones, and granule proteins- are recognized as DAMPs that can trigger inflammation, inducing cell death and organ failure. Extracellular histones are elevated in patients with coagulopathy and multiple organ failure (186) and are believed to be a major mediator of death in sepsis (71). Cell free DNA has been shown to be cytotoxic and results in coagulopathy and disseminated intravascular coagulation (DIC) (33) Additionally, inhibition of NETosis via PAD4 deficiency or inhibition results in a reduction in the release of DNA and improves outcomes in sepsis (187–189).

Concomitantly, various DAMPs have been shown to induce NETosis. Tadie et al. demonstrated that HMGB-1 is able to induce NETosis via TLR4 signaling (190). Incubation of neutrophils with HMGB-1 resulted in increased extracellular DNA, histone 3, and histone 3 citrullination. Exposure of neutrophils isolated from wild type and RAGE KO mice to HMGB1 resulted in significant NET formation, whereas neutrophils from TLR4 KO mice demonstrated a diminished ability to form NETs. Finally, HMGB1 acted synergistically with LPS, as neutrophils from the bronchoalveolar lavage (BAL) of mice exposed to both LPS and HMGB1 displayed greater ability to produced NETs compared to neutrophils isolated from the BALs of mice that received LPS alone. This increase was hindered by a neutralizing antibody to HMGB1 (190).

eCIRP has also been shown to activate NETosis through a TLR4/NF-κβ dependent mechanism (140). Mice subjected to polymicrobial sepsis via cecal ligation and puncture demonstrated increased levels of ICAM-1^+^ neutrophils in both the blood and the lungs. In contrast, mice genetically deficient in CIRP displayed diminished levels of ICAM-1^+^ neutrophils. *In vitro*, treatment of neutrophils with recombinant murine CIRP (rmCIRP) increased levels of ICAM-1^+^ neutrophils, and this increase was inhibited by both a neutralizing antibody to TLR4 or an NF-κβ inhibitor. ICAM-1^+^ neutrophils displayed increased levels of NETosis (140).

Unlike eCIRP and HMGB1, mitochondrial DNA (mtDNA) seems to generate NETosis through a TLR9 dependent pathway. mtDNA induced NADPH oxidase-independent NET formation in polymorphonuclear neutrophils of healthy volunteers. NETosis was completely inhibited by treatment with a TLR9 inhibitor (130). Liu et al. further identified that mtDNA also activates NETosis via the STING pathway (191). Neutrophils treated with mtDNA demonstrated increased NETosis in a manner which displayed significant increases of AKT and ERK1/2 phosphorylation and increased expression of Rac2 and PAD4. They further confirmed that both TLR9 and STING pathways are important in mtDA-induced NETosis via examination of the lungs of mice intravenously injected with mtDNA (191). Lungs displayed decreased NET formation in TLR9 KO and STING KO mice compared to wild type mice. Additionally, *in vitro* stimulation of BMDN from TLR9^−/−^ and STING^−/−^ mice displayed decreased percentages of NETs after treatment with mtDNA as compared to WT mice (191). Further confirming that mtDNA-induced NETosis proceeds through the Raf/MEK/ERK and p38 MAPK pathways, TLR9^−/−^ and STING^−/−^ neutrophils exhibited decreased phosphorylation of ERK 1/2 and p38 MAPK, as well as decreased levels of PAD4 and Rac2 after stimulation with mtDNA than WT neutrophils did. Inhibitors of these downstream mediators resulted in decreased mtDNA-induced NET formation in WT neutrophils (191).

Oxidized low-density lipoproteins (oxLDL) are upregulated in sepsis and intestinal inflammation (192) and have been recognized as a DAMP (193). *In vitro* treatment of PMNs with oxLDL resulted in increased NET formation in a dose dependent manner. oxLDL stimulation of NETosis seems to depend on TLR2 and 6; blocking of neutrophils with a TLR4 antibody had no effect on NET formation, while blocking with anti-TLR2 or TLR6 antibodies modestly reduced NETosis. However, the combination of anti-TRL2 and anti-TLR6 antibody treatment of PMNs prior to oxLDL stimulation resulted in a significant reduction in the formation of NETs (194). Additionally, confirming the role of the PKC pathway in oxLDL-induced NETOsis, inhibition of PKC or IRAK was able to reduce NET formation in normal neutrophils. Inhibition of downstream mediators in the pathway, ERK1/2 and p38 MAPK, also reduced oxLDL-induced NET formation (194).

## Future Directions and Conclusions

In this review article, we discussed DAMPs and NETs in sepsis, with a focus on their interaction and therapeutic strategies for amelioration of sepsis-associated morbidity and mortality. Future studies on the interaction between the two entities would add value to the study of innate immunology and could be expanded to other inflammatory conditions in addition to sepsis. Moreover, future emphasize should also be focused on pinpointing the relationship between PAMPs and NETs and developing new therapeutic tools to target their interplay. DAMPs are released by several cell types, while NETs are specific to neutrophils. Recently, extracellular traps (ETosis) has been described in macrophages (195) and eosinophils (196). Future studies on DAMPs and ETosis would be interesting. Immune cells in sepsis are very plastic with several phenotypic polarizations—more investigation is needed into the role of immune cell plasticity on DAMP release. Similarly, future studies on how DAMPs skew immune cell polarization and the subsequent impact on sepsis would be revealing. In conclusion, we have provided a literature review of the role of DAMPs, NETs, and their interaction in sepsis to increase and update our understanding in this area of research.

## Author Contributions

N-LD and MA did literature review and wrote the manuscript. SG helped in writing the extracellular DNA section and reviewing the manuscript. N-LD prepared the table and MA prepared the figures. PW reviewed, edited the manuscript, and conceived the original idea of the project.

### Conflict of Interest

The authors declare that the research was conducted in the absence of any commercial or financial relationships that could be construed as a potential conflict of interest.
